# Differences in Memory, Perceptions, and Preferences of Multimedia Consumer Medication Information: Experimental Performance and Self-Report Study

**DOI:** 10.2196/15913

**Published:** 2020-12-01

**Authors:** Helen Monkman, Andre W Kushniruk, Elizabeth M Borycki, Debra J Sheets, Jeffrey Barnett

**Affiliations:** 1 School of Health Information Science University of Victoria Victoria, BC Canada; 2 School of Nursing University of Victoria Victoria, BC Canada

**Keywords:** consumer medication information, medication guides, patient medication information, prescription drug information leaflet, patient information leaflets, multimedia learning, health literacy, eHealth literacy, consumer health informatics, cognitive theory of multimedia learning

## Abstract

**Background:**

Electronic health resources are becoming prevalent. However, consumer medication information (CMI) is still predominantly text based. Incorporating multimedia into CMI (eg, images, narration) may improve consumers’ memory of the information as well as their perceptions and preferences of these materials.

**Objective:**

This study examined whether adding images and narration to CMI impacted patients’ (1) memory, (2) perceptions of comprehensibility, utility, or design quality, and (3) overall preferences.

**Methods:**

We presented 36 participants with CMI in 3 formats: (1) text, (2) text + images, and (3) narration + images, and subsequently asked them to recall information. After seeing all 3 CMI formats, participants rated the formats in terms of comprehensibility, utility, and design quality, and ranked them from most to least favorite.

**Results:**

Interestingly, no significant differences in memory were observed (*F*_2,70_=0.1, *P*=0.901). Thus, this study did not find evidence to support multimedia or modality principles in the context of CMI. Despite the absence of effects on memory, the CMI format significantly impacted perceptions of the materials. Specifically, participants rated the text + images format highest in terms of comprehensibility (χ^2^_2_=26.5, *P*<.001) and design quality (χ^2^_2_=35.69, *P*<.001). Although the omnibus test suggested a difference in utility ratings as well (χ^2^_2_=8.21, *P*=.016), no significant differences were found after correcting for multiple comparisons. Consistent with perception findings, the preference ranks yielded a significant difference (χ^2^_2_=26.00, *P*<.001), whereby participants preferred the text + images format overall. Indeed, 75% (27/36) of participants chose the text + images format as their most favorite. Thus, although there were no objective memory differences between the formats, we observed subjective differences in comprehensibility, design quality, and overall preferences.

**Conclusions:**

This study revealed that although multimedia did not appear to influence memory of CMI, it did impact participants’ opinions about the materials. The lack of observed differences in memory may have been due to ceiling effects, memory rather than understanding as an index of learning, the fragmented nature of the information in CMI itself, or the size or characteristics of the sample (ie, young, educated subjects with adequate health literacy skills). The differences in the subjective (ie, perceptions and preferences) and objective (ie, memory) results highlight the value of using both types of measures. Moreover, findings from this study could be used to inform future research on how CMI could be designed to better suit the preferences of consumers and potentially increase the likelihood that CMI is used. Additional research is warranted to explore whether multimedia impacts memory of CMI under different conditions (eg, older participants, subjects with lower levels of health literacy, more difficult stimuli, or extended time for decay).

## Introduction

### Background

Facilitating consumers to find, assess, and understand health information and to make effective decisions based on that information is the impetus for research on health literacy [1]. Further, the increasing availability of online and digital health information motivates a similar need to study digital or eHealth literacy [2]. eHealth literacy is “the ability to seek, find, understand, and appraise health information from electronic sources and apply the knowledge gained to addressing or solving a health problem” [2]. Digital media (eg, internet, mobile apps) have the potential to create new opportunities and streamline information for consumers (eg, tailoring, progressive disclosure). However, they also have the potential to create additional challenges for consumers trying to find and use health information, so the design of the system and how the information is written require careful consideration and study.

One example of consumers requiring health information is when they take medications. Approximately, 4 in 10 Canadians (40.5%) between the ages of 6 and 79 years take at least 1 prescription medication and, unsurprisingly, people are more likely to take medications the older they are [3]. Given the widespread use of prescription medications, consumers should understand and remember information about the medications they take in order to maximize the therapeutic benefits and minimize the risks. Moreover, memory (ie, recalling or recognizing information) and comprehension (ie, understanding information) are factors proposed to affect therapy adherence [4,5]. In this study, we are emphasizing the importance of recalling information about the medication (eg, administration, storage, side effects) as opposed to remembering the particular time to use a medication—a distinct area warranting research. Essentially, by providing consumers with medication information, we are trying to help them understand how to take the medication, what to avoid, what to watch out for, etc. A systematic review of written medication information indicated that consumers appreciate and use this information and it may improve medication adherence [6].

Methods of communicating medication information to consumers need to be carefully studied, designed, and deployed. Relying solely on verbal communications for medication information is not prudent because memory is generally poor. Specifically, consumers only remember 20% to 60% of information that health care professionals discuss verbally immediately after the interaction [7-9]. Therefore, it is important to supply complementary and supplementary information to consumers to improve comprehension, memory, and ideally adherence and therapeutic benefits while minimizing risks. Moreover, merely providing long text-only handouts may not encourage consumers to read, understand, and remember the information.

It is important to explore materials that offer more than simply text to determine the impact of visuals and potentially narration to create more appealing and robust representations of medication information. Many studies have shown that multimedia benefits learning, and there are principles guiding how multimedia can be most effectively applied [10-12]. For example, it may be worthwhile exploring the use of data visualizations for communicating the likelihood of side effects rather than merely relying on vague terms such as “possible” or “common.” There are a variety of worthwhile avenues for exploration to improve medication information beyond what is currently available. This study used a common consumer resource for medication information (ie, consumer medication information [CMI]) and systematically transformed it using multimedia (ie, added images to text, replaced text with narration) to determine the effect of incorporating multimedia on memory, perceptions of comprehensibility, utility, and design quality, as well as overall preference.

### CMI

CMI attempts to address the need for medication information that can be subsequently referenced. CMI, for the purposes of this study, is the term used for the text-based paper information sheet(s) typically given to consumers at Canadian pharmacies when a prescription is filled for the first time. Although there is guidance for CMI, it is not regulated by Health Canada and unfortunately, as a result, there are often considerable disparities between CMI sourced from different pharmacy chains [13].

CMI contains typical information about what the medication is used to treat and its common dosage, but it may not match the individual consumer’s actual prescription or condition. CMI conveys a variety of general information about the medication including the following: dispensing pharmacy (eg, name, address, phone number), consumer’s name, prescriber’s name, date, brand and chemical (or generic) names of the medication, drug identification number (DIN), conditions that the medication is usually used to treat, how the medication is typically administered, potential side effects, important information about the medication, and how to store the medication.

Many posit that, as currently designed and delivered, CMI and other similar types of medication information offer limited value to users. Findings from a review on written medication information suggests that its value is currently limited because of language complexity, poor visual presentation, lack of tailoring, and use of words rather than numbers to convey risk of side effects [14]. Others have argued that medication information is often difficult to read and not suitable for consumers, especially older people [15] or those with limited health literacy [13,16-18]. Moreover, medication information may not be adequately addressing user information needs by failing to provide answers to questions consumers want to know about their medications [6]. Although we are generally seeing a shift from hard copy materials to digital options or replacements, this has not yet been observed with respect to CMI. However, when this shift inevitably occurs, we should be prepared with evidence to inform the design and deployment of these materials to optimize consumers’ learning and use of them.

### Multimedia

Multimedia is an approach to information design that has yet to be systematically applied and investigated for its potential benefits in disseminating health information to consumers [19]. Multimedia research is motivated by evidence that combining multiple methods of communication to convey information is more successful than relying on a single method. Thus, the definition of multimedia is “presenting words (such as printed text or spoken text) and pictures (such as illustrations, photos, animation, or video)” [12]. Domains such as education, entertainment, advertising, and more recently health care have embraced the benefits of multimedia [20]. Additionally, investigations of the potential benefits of multimedia for communication of health information [21], and even medication information specifically [22], for consumers have begun. However, these studies have largely overlooked the body of research done in multimedia learning and therefore the materials developed may not be as effective as possible [19].

Mayer [10] developed the cognitive theory of multimedia learning (CTML) to integrate the evidence and depict how people process multimedia presentations. Effects consistently observed and reported in multimedia learning studies have been organized into a set of multimedia principles that are used to both (1) describe why particular cognitive phenomena occur and (2) guide multimedia design to ensure it is done most effectively [10-12]. Thus, it is important to leverage existing evidence-based multimedia principles for the design of new multimedia health information to optimize its efficacy [19].

Given its demonstrated benefits in other domains, multimedia is a promising method of enhancing understanding and memory of medication information. There are many multimedia principles and new ones are continuously being developed [12]. However, this study only explored the following multimedia and modality principles: (1) people learn better from words and images than words alone [10], and (2) people learn better from narration and images than from written words and images [10].

### Motivation and Research Questions

There are emerging studies that are attempting to improve CMI and other medication information for consumers. However, there were 4 primary factors that were not adequately addressed in other studies that motivated this study: (1) the failure to isolate the effect of multimedia, (2) the limited use of multimedia in stimuli, (3) the exploration of possible multimedia effects for younger people with adequate health literacy, and (4) the dearth of studies examining narration.

First, most previous studies that explored potential opportunities to improve different types of medication information have manipulated multiple aspects of design and content simultaneously. Moreover, most of the recent research seeking to improve medication information for consumers has concentrated on modifying both its content and its layout. There is evidence that various layout redesigns (eg, 2 columns, segmented sections, modelled after over-the-counter drug facts boxes [23]) improve consumers’ perceptions of medication information, such as ratings of comprehensibility [24], utility, or design quality, or all 3 [25], as well as ease of locating information [26], attractiveness, readability [27], attitude toward the materials, and intention to read it [28]. In addition to increasing consumers’ subjective ratings, layout redesigns have also bolstered different aspects of performance, such as locating information more quickly and effectively [24,29], as well as improving comprehension [24,27-30]. However, a major shortcoming of these studies is that the redesigned layouts was paired with changes in the length of the materials. Thus, the content was not controlled and instead were also modified in conjunction with layout. Therefore, comparisons were often between lengthier (control or current practice) and briefer stimuli, which confounded their results. For example, one study [30] compared a 4-page medication guide with a 1-page redesign. Thus, it is not necessarily surprising that consumers understood the shorter materials better, as there was less information that could potentially distract them or exceed their cognitive processing capabilities. Similarly, studies that have added multimedia to medication information typically made modifications to content as well [28,31]. For example, in addition to adding icons to represent dosing schedule, one study also increased the font size, lowered readability scores, and shortened and reorganized the content [31]. Again, the impact of multimedia cannot be distinguished from the effects of other modifications to the stimuli.

Investigations such as those above are valuable because they demonstrate that design and content changes can improve perceptions (eg, ratings of comprehensibility, utility, design quality, attractiveness, readability, attitudes and intentions) and performance (eg, comprehension, memory, information location) of medication information. However, by changing multiple aspects of the stimuli simultaneously, their methods preclude attributing gains to individual factors (eg, multimedia, length, readability, organization, layout). In contrast, this study used the same content for all 3 formats to determine if multimedia affected memory, perceptions, and/or preferences. That is, the exact same words and sequence of words were used to describe a medication, regardless of whether its presentation format (ie, text, text + images, or narration + images). This control allowed for the potential effect of multimedia to be isolated.

Second, studies exploring the impact of multimedia on medication information have generally limited the use of images to complement text to a narrow component of medication information, such as dosing schedules [31,32], directions and precautions [33], or only a few symbols and an image of the medication itself [28]. Thus, to address this shortcoming in the existing literature around use of multimedia medication information, this study included images throughout the entire presentation (eg, indications, side effects).

Third, most studies have focused their efforts on improving medication information using multimedia for particular groups of people who may inherently have more difficulty processing this information and therefore may have the most to gain. Specifically, multimedia medication information has been explored for older people [31,34] and people with limited health literacy [33,35]. However, it is also worthwhile to determine if multimedia benefits people who do not belong to these groups.

Fourth, no studies were identified that have explored the use of narration for medication information specifically. In response, the proposed study created a format of CMI using narration to convey information in lieu of text with complementary images.

### Research Questions and Approach

This study examined memory, perceptions, and preferences by investigating the following 7 research questions: (1) Is there evidence of a multimedia effect for CMI on memory (ie, does adding images to text impact memory for CMI)?, (2) Is there evidence of a modality effect for CMI on memory (ie, does using narration instead text accompanied by images impact memory for CMI)?, (3) Are there differences in how participants perceive the CMI formats in terms of comprehensibility?, (4) Are there differences in how participants perceive the CMI formats in terms of utility?, (5) Are there differences in how participants perceive the CMI formats in terms of design quality?, (6) Do most participants’ share a favorite CMI format?, and (7) Do most participants’ share a least favorite CMI format?

We used an objective approach to investigating participants’ memory and a subjective approach to determining their perceptions and preferences regarding CMI in 3 formats: text, text + images, and narration + images. We tested participants’ memory by having them respond to free recall questions for each CMI format. We determined perceptions by having participants rate the CMI in terms of comprehensibility, utility, and design quality. Finally, participants ranked the 3 formats from most to least favorite to indicate overall preference.

## Methods

### Sample Size Calculation

The number of participants needed to achieve a significant difference between conditions in this study was estimated based on findings from a meta-analysis comparing the effectiveness of static images versus animations [36]. Höffler and Leutner [36] found that the mean weighted effect size was 0.44 for declarative knowledge (ie, memory) in 40 studies. Thus, to calculate the number of participants for the proposed study, we adopted a critical effect size of 0.45, significance level of 0.05, and power of 0.8. Using the aforementioned parameters for 1-tailed tests, as memory hypotheses were directional, a sample size of 28 participants was suggested [37]. However, given the counterbalancing, we needed a number divisible by 6, and to be even more conservative (ie, run 1 more participant in each sequence than suggested), 36 participants were recruited for this study.

### Recruitment and Remuneration

To advertise the study, the investigators sent out a call for participants through the University of Victoria’s School of Health Information Science listserv and hung posters on campus to advertise the study. Each participant received a gift card worth Can $20 (US $15.38) as compensation for their time.

### Participant Exclusion

Participants were excluded by self-report from the study for any of the following reasons: (1) they had a medical or health professional background (eg, nurses, pharmacists, doctors), (2) they were not proficient in the English language, or (3) they had compromised visual or auditory acuity that was not effectively compensated for by assistive devices (eg, glasses, hearing aids).

Two participants were identified as outliers due to their age (ie, >3 SD from the mean age) and replaced with 2 new participants to maintain equal numbers of participants in each sequence.

### Materials

#### Stimuli Selection

Two authors (HM and JB) generated a list of 23 medications to consider for use as stimuli. Possible CMI stimuli were collected and reviewed from a leading community pharmacy chain. The investigators transcribed and compared the CMI based on the conditions that the medications treated and routes of medication administration, as well as the length (ie, number of words) and readability of the materials. Three medications (Betaderm [Taro Pharmaceuticals Inc], cromolyn, and Flovent [GlaxoSmithKline]) were selected based on their uniqueness from each other in terms of name, route of administration, and informational content, as well as the similarity in the length and readability of their CMI.

#### CMI Formats

Three different CMI formats served as conditions in this study: text, text + images, and narration + images. Three health care professionals (2 nurses and 1 pharmacist) reviewed the final materials to ensure that they were representative of typical CMI, a technique used in other studies to validate stimuli [38]. The following sections will describe in more detail how we developed the 3 different formats.

#### Text Format (Control)

The text format served as the control condition for this experiment because it closely resembled CMI that consumers currently receive from Canadian pharmacies. We transcribed the content from the CMI of a leading community pharmacy and simplified it slightly to create the text format. Specifically, the date, DIN, address, and phone number of the community pharmacy, as well as other branding and logos, were excluded from the text format. Additionally, the “general information” section and “storage” instructions for the CMI were excluded, as they were nearly or virtually identical for all 3 medications. Therefore, these 2 topics provided no unique learning opportunities that would be more likely to be remembered in subsequent conditions because of repeated exposure. We used Arial 12-point font throughout, and headings were bolded (see Multimedia Appendix 1).

#### Text + Images Format

The text + images format was developed by complementing the text format with images from the internet (see Multimedia Appendix 2). The page layouts were 11 inches in width and as long as necessary to convey all of the information. As in the text format, Arial font was used. However, larger font sizes were used (ie, 14-point font for body text and 22-point font for medication names) for the text + images format. We made minor changes to punctuation (eg, removing periods), added a few words (eg, the name of the condition next to the picture of the condition), replaced written numbers with Arabic numerals, emphasized medication names and headings, and used boxes to group topic information. However, the content (ie, words) in the text + images format remained identical to that in the text format. Text + images formats were saved as PDF files.

#### Narration + Images Format

We generated the narration + images format by adding an audio recording of a volunteer reading the text format aloud and using the images from the text + images format. The narration + images format was a series of narrated PowerPoint (Microsoft Inc) slides using the same font and image sizes as the text + images format. However, the font size was reduced during exposure, as a result of the width available for showing the video in the survey software. Very few select words were retained if they were considered to frame the presentation (eg, the name of the medication, headings) or to reinforce the meaning of images (eg, names of side effects). The narrated PowerPoint presentation was screen recorded with audio and played for participants via YouTube (see Multimedia Appendix 3).

### Apparatus

We gave participants hard copies of the text format on 8.5×11-inch paper to emulate the current dispensing practice of CMI at Canadian pharmacies. We displayed the remaining 2 formats (ie, text + images and narration + images) on an Apple Macbook Air laptop computer with a 13.3-inch colour display. The text + images format was displayed on a single webpage (scrolling required). Participants were shown the narration + images format as an embedded YouTube video. To keep the exposure timing consistent, participants were only able to watch the video once from start to finish. We recorded the computer screen and audio using QuickTime media player (Apple Inc), even when the computer was not involved (eg, when participants were studying text format) and made an additional audio recording using a digital recorder.

### Setting

The experiment was conducted in a quiet office. Participants were seated comfortably at a desk and the experimenter sat alongside him or her with the experimental materials that were not currently in use (eg, text format).

### Procedure and Measures

#### Experimental Design

This experiment used a 1×3 randomized, counterbalanced design. The single factor (ie, independent variable) was CMI format and the 3 levels of CMI format were text, text + images, and narration + images. This study design was used to investigate the potential effect of multimedia CMI on memory, perceptions (ie, comprehensibility, utility, and design quality), and overall format preference.

**Figure 1:**
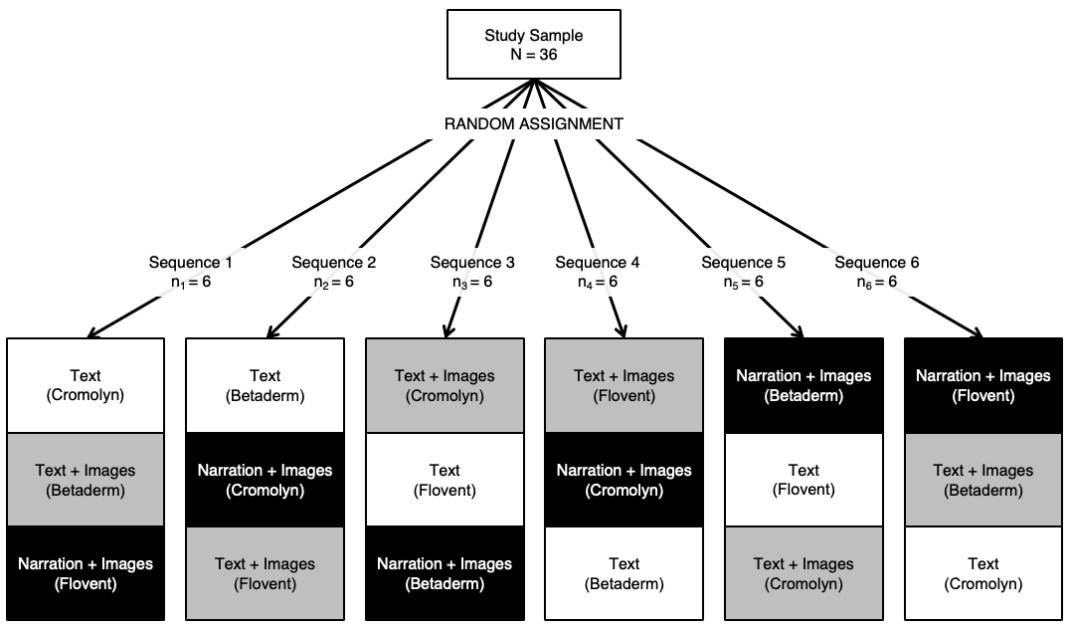
Experimental design: randomized and counterbalanced for format and medication (in brackets).

All 36 participants were randomly assigned to 1 of the 6 unique presentation sequences counterbalancing for CMI format and medication (Figure 1). At the onset of each session, the participant pulled a number from a container to select the presentation sequence, which then dictated the order of CMI format and which medications were shown in each format. To ensure equal cell sizes, numbers were drawn without replacement.

The presentation sequences (Figure 1) determined the 3 conditions (ie, the unique combinations of format and medication). Thus, participants saw all 3 formats and a different medication in each format. The order of both the CMI format and the medications were counterbalanced. We took these precautionary measures in an attempt to minimize the potential for order effects, fatigue effects, and inherent memorability differences between medications.

#### Procedure

After reading and signing the informed consent form, each participant drew a piece of paper with a number on it from the container, determining his or her sequence. Next, the participants completed preliminary measures for descriptive purposes. Specifically, we administered a demographic questionnaire, the Newest Vital Sign (NVS) [39], and the eHealth Literacy Scale (eHEALS) [40].

Following administration of the preliminary measures, the procedure was identical (with the exception of the stimulus) for each of the 3 experimental trials. We adapted the experimental trial procedure from methods used by Morrow and colleagues [31] and encouraged participants to create mental models by asking them to try and understand the medication information rather than simply memorize it [41]. For each of the 3 trials, the following steps occurred:

1. Stimulus exposure: first, participants saw a condition (ie, CMI format and medication combination determined by the presentation sequence). The narration + images format ranged from 1 minute 57 seconds to 2 minutes 12 seconds. Participants saw the text and text + images formats for up to 2 minutes as well. Participants were able to move on to the next step before the time elapsed.
2. Distractor task: participants then completed a slightly modified version of the adapted Consumer Information Rating Form (CIRF) [42] as a distractor task to prevent rehearsal of the information and allow time for information to decay from memory. Additionally, the CIRF [42] familiarized the participants with the concepts of comprehensibility, utility, and design quality.
3. Memory task: the investigator then asked participants to recall information about the medication aloud (see Multimedia Appendix 4 ).

The aforementioned 3 steps were repeated until participants saw all 3 conditions (ie, all 3 formats and all 3 medications).

After completing the third and final experimental trial (ie, after having seen all 3 conditions), participants indicated their overall perceptions of the 3 CMI by rating each of the 3 formats on 3 dimensions: comprehensibility, utility, and design quality (see Multimedia Appendix 4). The 3 perception dimensions were based on the subscales of the adapted CIRF [42]. Participants then indicated their preferences by ranking the formats from most to least favorite; ties were not permitted (see Multimedia Appendix 4).

### Analysis

#### CMI Memory

The audio recordings from the study were transcribed in full. The method of assessing memory was adopted from another study [43]. Specifically, each content item correctly generated by the participant that matched a CMI content item (ie, individual item of information, such as a side effect) was awarded a mark. Points were only awarded once for synonyms (eg, “topical” or “applied to the skin”) or for information that was repeated in the CMI (eg, prescription strength). However, the 3 medications did vary slightly in terms of the total number of content items. Specifically, Betaderm had 28 content items, cromolyn had 29, and Flovent had 28.

Omnibus analysis of variance (ANOVA) analyses were conducted on participants’ memory scores to investigate whether the CMI format influenced memory. When the omnibus tests were significant, we made pairwise comparisons. A between-groups ANOVA explored potential memory differences in the first condition to avoid any potential influence of practice effects. A repeated-measures ANOVA determined whether memory was affected by CMI format across all 3 conditions.

#### Perceptions and Preference Comparison

Participants rated the 3 CMI formats on each of the 3 perceptual constructs (ie, comprehensibility, utility, and design quality) and ranked them from most to least favorite. Given the ordinal nature of the data, a series of nonparametric Friedman tests of difference among repeated measures were conducted to investigate whether participants rated CMI formats differently in terms of comprehensibility, utility, design quality, and overall preference. Where Friedman tests were significant, pairwise Wilcoxon signed rank tests were used for pairwise comparisons. Post hoc analysis with Wilcoxon signed rank tests was conducted with a Bonferroni correction applied (α=.05/3), resulting in a significance level set at *P*<0.017.

## Results

### Participant Characteristics

A summary of the participants’ characteristics (demographic, educational, and medication related) can be found in Table 1. The mean age of the participants was 23.6 years (SD 3.8; range 18-35). Most participants in this study were female (26/36, 72%), identified as Caucasian (23/36, 64%), and reported English as their first language (31/36, 86%). All of the participants were students. The majority of participants were currently enrolled in school full-time (30/36, 83%). Participants were students of various faculties, but the 3 most common faculties were science (9/36, 25%), social sciences (8/36, 22%), and human and social development (7/36, 19%).

Participants reported using several different resources for medication information. The most commonly reported medication resources were physicians (27/36, 75%). An equal number of participants reported consulting pharmacists (16/36, 44%) and electronic resources (16/36, 44%) for information about medications. Many participants (16/36, 44%) reported not taking any prescription medications daily; however, over one-third (13/36, 36%) of participants reported taking 1 medication daily. Nearly one-half (17/36, 47%) of the participants reported following medication instructions completely.

**Table 1:** Participants’ characteristics (N=36).

Characteristics		Frequency, n (%)
**Gender**		
	Female	26 (72)
	Male	10 (28)
**Ethnicity**		
	Caucasian	23 (64)
	Asian	10 (28)
	Other ethnicity	1 (3)
	Multiple ethnicities (ie, 2 or more reported)	3 (8)
**First language**		
	English	31 (86)
	Other	5 (14)
**School enrollment status**		
	Full-time	30 (83)
	Part-time	3 (8)
	Cooperative education	3 (8)
**Faculty of study**		
	Science	9 (25)
	Social science	8 (22)
	Human and social development	7 (19)
	Education	4 (11)
	Other (eg, business, engineering, fine arts, law)	8 (22)
**Medication information resources consulted^a^**		
	Physician	27 (75)
	Pharmacist	16 (44)
	Electronic resources (eg, internet)	16 (44)
	Family member	9 (25)
	Other	1 (3)
**Number of prescription medications taken daily**		
	0	16 (44)
	1	13 (36)
	2	6 (17)
	3	1 (3)
**Follow medication instructions**		
	Completely	17 (47)
	Mostly	9 (25)
	Somewhat	8 (22)

^a^Sum exceeds 100% because participants could report using multiple medication resources.

According to Weiss and colleagues’ marking framework [39], most participants (30/36, 83%) were likely to have adequate health literacy. Six participants (17%) were classified as possibly having limited health literacy. However, no participants had a high likelihood of limited health literacy. Interestingly, using Monkman and colleagues 4-category framework [44] to classifying self-perceptions of eHealth literacy using eHEALS [40] scores, only a minority (8/36, 22%) of participants had high eHealth literacy scores [44]. The majority of participants reported only moderate (21/36, 58%) self-perceptions of eHealth literacy [44]. Concerningly, 7 participants (19%) reported low self-perceptions of eHealth literacy [44]. However, no participants lacked self-perceived eHealth literacy skills [44]. Interestingly, there was no correlation between participants’ scores on the NVS [39] and the eHEALS [40], calling into question the extent of the relationship between health literacy and eHealth literacy or the respective measures used [44].

### Effects of Multimedia on Memory of CMI

First, to negate any practice effects (eg, studying and rehearsing answers specific to recall questions), participants’ memory in the first condition was examined. A 1-way, between-subjects ANOVA yielded no indication of CMI format affecting memory (*F*_2,33_=0.19, *P*=.830). Mean number of items remembered on participants’ first attempt with the memory task was 12.00 (95% CI 9.64-14.36; range 5-17) for the text format, 11.25 (95% CI 9.39-13.11; range 7-17) for the text + images format, and 11.75 (95% CI 10.24-13.26; range 9-16) for the narration + images format. Second, to minimize the effect of individual differences (eg, some participants having better memories), participants’ memory in all 3 conditions was compared. Again, a 1-way, repeated-measures ANOVA determined there was no significant effect of CMI format on memory (*F*_2,70_=0.1, *P*=0.901). The mean number of items remembered in the memory task for all participants was 12.44 (95% CI 11.05-13.84; range 5-25) for the text format, 12.53 (95% CI 11.28-13.78; range 6-21) for the text + images format, and 12.75 (95% CI 11.71-13.79; range 7-218) for the narration + images format.

In summary, there was no evidence to support either the multimedia principle or the modality principle. That is, participants remembered approximately the same amount of information regardless of whether the CMI was presented as text, text + images, or narration + images in the first condition and across all 3 conditions.

### Comparison of Participants’ Perceptions and Preferences of CMI Formats

All 3 Friedman tests comparing participants’ perceptions of the 3 CMI formats were significant. Specifically, the Friedman tests yielded comprehensibility (χ^2^_2_=26.5, *P*<.001), utility (χ^2^_2_=8.21, *P*=.016), and design quality (χ^2^_2_=35.69, *P*<.001). Post hoc analyses with Wilcoxon signed-rank tests with a Bonferroni correction applied resulted in a significance level set at *P*<0.017. These pairwise comparisons indicated that participants rated the text + images format higher than both the text format and the narration + images format in terms of comprehensibility and design quality (Table 2). Further, narration + images was also rated significantly higher than the text format on these 2 dimensions. Despite the significant utility omnibus test, differences between the pairwise comparisons did not reach the threshold for significant differences (Table 2).

**Table 2:** Summary of pairwise Wilcoxon signed-rank tests for perception and preference ratings.

Perceptual dimension	Pairwise comparison	Standardized test statistic	*P* value	Significant difference at *P*<.017?
Comprehensibility	Text, text + images	–4.27	<.001	Yes
	Text, narration + images	–3.11	.002	Yes
	Narration + images, text + images	–2.61	.009	Yes
Utility	Text, text + images	–2.32	.021	No
	Text, narration + images	–1.18	.236	No
	Narration + images, text + images	–2.17	.030	No
Design quality	Text, text + images	–4.53	<.001	Yes
	Text, narration + images	–4.50	<.001	Yes
	Narration + images, text + images	–2.53	.011	Yes
Overall preference ranking	Text, text + images	–4.20	<.001	Yes
	Text, narration + images	–1.57	.116	No
	Narration + images, text + images	–3.72	<.001	Yes

#### Overall Preference Ranking

The majority of participants selected the text + images format as their most favorite (27/36, 75%) and the text format as their least favorite (23/36, 64%). A Friedman test of difference comparing participants’ rankings of the 3 CMI formats revealed that this pattern was significant (χ^2^_2_=26.00, *P*<.001). Again, to account for multiple comparisons, a Bonferroni correction was applied to the Wilcoxon signed-rank tests, setting the threshold of significance to *P*<0.017. The text + images format was preferred overall to both the text format and the narration + images format. However, there was no significant difference in preference between the text and narration + images formats (see Table 2).

## Discussion

### Principal Results

This study sought to determine whether multimedia CMI impacted memory, perceptions, and/or preferences for CMI. A summary of the findings to the specific research questions posed at the onset of this experiment can be found in Table 3. The use of multimedia (ie, images, narration) in CMI did not appear to have any influence on memory in this experiment. Despite the lack of evidence to support any differences in memory between the CMI formats, there were observable differences in participants’ perceptions of and preferences for the 3 CMI formats (see Table 3). Specifically, the text + images format was rated the highest in terms of design quality and comprehensibility and was also selected by the participants’ most frequently as their most favorite CMI format.

**Table 3:** Summary of research areas, questions, and findings.

Research area and question		Supported (yes or no)?	Finding
**Memory**			
	Is there evidence of a multimedia effect for CMI^a^ on memory?	No	No differences in memory were observed between the text and text + images formats.
	Is there evidence of a modality effect for CMI on memory?	No	No differences in memory were observed between the text + images and narration + images formats.
**Perceptions**			
	Do participants perceive one CMI format as more comprehensible?	Yes	Participants perceived the text + images format as the most comprehensible.
	Do participants perceive one CMI format as having more utility?	Mixed	The omnibus test was significant but there were no significant differences between the 3 formats after adjusting for pairwise comparisons.
	Do participants perceive one CMI as superior in terms of design quality?	Yes	Participants perceived the text + images format as the most comprehensible.
**Preferences**			
	Do most participants share a most favorite CMI format?	Yes	Most participants selected the text + images CMI format as their most favorite and it ranked significantly higher than both the text and narration + images formats.
	Do most participants share a least favorite CMI format?	Mixed	Most participants ranked the text format as their least favorite, but there was no significant difference between the narration + images and text format rankings.

^a^CMI: consumer medication information.

### Memory Results

Participants remembered approximately the same amount of information, regardless of what CMI format they saw and thus there was no evidence to support the multimedia or modality principles in this study. If the multimedia and modality effects were observed, the expected pattern of results would have been that participants remembered the most in the narration + images condition, followed by the text + images condition, and the least in the text condition. The results from this study suggest that the CTML [10-12] does not apply to CMI, at least with respect to memory performance as an index of learning. Similarly, King et al [33] failed to show significant effects of multimedia on memory for medication information. Although their study limited their test stimuli to medication directives (ie, directions and precautions) [33], this study used multimedia to complement as much of the written content in CMI as possible. Additionally, this study also investigated whether narration had an impact on CMI memory, which failed to generate differences either.

Do these findings (or more accurately lack thereof) insinuate that developing multimedia materials for CMI and consumer health information is a poor investment? Despite the lack of evidence to support previous assertions promoting the importance of multimedia in consumer health information [19], multimedia may still in fact be very valuable in consumer health communications. There are several reasons why multimedia consumer health information warrants continued investigation: memory ceiling effects; memory, not understanding, as an index of learning; CMI is a fragmented description, not a narrative process explanation; multimedia benefits some more than others; and multimedia improves perceptions and people prefer it.

#### Memory Ceiling Effects

It is possible that we observed a ceiling effect in memory performance in this study. A ceiling effect occurs when the dependent variable values are all near their maximum [45] and as such, the manipulation of the independent variable cannot result in additional gains. Performance on the memory task in this study was quite high even on the first trial, with means ranging from 11.25 to 12.00 on individual CMI items. Thus, it is possible that the experimental design (eg, stimuli content length and complexity, distractor task) did not have conditions challenging enough to create observable differences in memory due to multimedia. This finding is positive in that it indicates that people can recall much of the information contained in CMI if they study it. However, the CMI used in this study, from a leading pharmacy in Canada, was deemed to be the most “patient-centered” (ie, brief, with bullet points), and therefore these findings may not apply to CMI that is longer and/or more complex. Additionally, the distractor task was not a typical verbal interference task (eg, crossing out e’s in a written passage as used by Morrow et al [31]. The CIRF [42] was used as a more naturalistic task to have participants reflect on the strengths and weaknesses of the CMI and simultaneously allowing time for potential memory decay. It would be valuable to repeat this study using more complex stimuli and potentially a different distractor task to determine if more variability in CMI memory performance can be observed under different experimental conditions.

#### Memory, Not Understanding, as an Index of Learning

The absence of expected learning gains due to multimedia may be attributable to this experiment only testing memory and not understanding. As previously described, the 2 primary goals of multimedia instruction are for learners to remember and understand [12]. Mayer [12] defined remembering as the “ability to reproduce or recognize presented material,” whereas understanding is the “ability to use presented material in novel situations.” Gains in performance due to multimedia appear to be consistent for understanding but variable for memory. Some studies have reported improved memory and comprehension due to multimedia presentations [46], yet others have found no benefits to memory, only to understanding [47-49]. Thus, perhaps because we only tested memory and not understanding, we failed to find any impact of the multimedia CMI formats. However, unlike some other consumer health information, CMI poses unique challenges to disambiguating memory from understanding and developing a valid comprehension test for medication information.

It is difficult to test for comprehension of medication information using CMI stimuli because CMI is inherently unique to each medication and it is rarely prudent to apply the knowledge about one medication broadly to a novel medication situation. Moreover, it is challenging to distinguish between what information consumers truly understand and what they simply remember. Although some researchers have reportedly tested understanding, they have only assessed memory. Indeed, Houts and colleagues [21] noted that several studies in their review “purported to assess comprehension but, in fact, studied recall since they only asked respondents to repeat information they heard or read.” Thus, it is not surprising that some researchers have conflated memory, understanding, and other cognitive abilities in medication information.

Similar studies [31,34] have used a valid, naturalistic comprehension test for prescription medication. However, it requires a dosing schedule from an individual’s prescription and because of the generic nature of CMI, it could not be used in this study. Specifically, the inference task charged participants to determine how many tablets would be consumed daily; thus, the participants had to calculate this value by multiplying how many tablets were taken each time and how many times a day they were taken [31]. Arguably, this inference task is a comprehension task, as it requires combining the information in a novel way to solve a problem. However, this task has limited value in the context of testing CMI, as CMI currently conveys only “typical” dosage frequency but not necessarily dosage amount. For example, CMI in this study indicated that the inhaler was typically used twice a day, but there was no information about how many puffs should be administered each time. The specific details of dose and time are prescribed uniquely, which often conveys more details and may vary further from what is descried in the CMI.

#### CMI is a Fragmented Description, Not a Narrative Process Explanation

The second possible explanation for why multimedia did not appear to affect memory for CMI is that CMI content may inherently be poorly suited for multimedia instruction because it requires learning discrete types of information. CMI is essentially a description of fragmented information (eg, indications, side effects, storage), whereby the topics are disconnected. In contrast, typical multimedia learning experiments explain processes (ie, sequences of events) such as how lightning works [50], the mechanics of pulleys [51], and the principles of flight [52]. In contrast, the stimuli in the present study were more descriptive than explanatory. That is, with the exception of medication instruction processes, most CMI is separated into discrete topics of information that would, from the consumers’ perspective, likely appear unrelated. This might also explain why King et al [33] failed to find any differences in memory associated with adding pictograms to medication information.

As previously described, it is difficult to test for CMI understanding, and CMI should generally not be used to make inferences. Mayer and Anderson [48] also noted how differences in content make information more or less suitable for multimedia instruction. Specifically, they described how the instructional material, or inherent characteristics of the stimuli, may play a role in multimedia learning: “we used materials that explained how a system works; that is, we focused on “how-it-works” explanations that could be used to make inferences. If we had focused on material consisting mainly of arbitrary facts, we would not have been able to test for understanding. In short, our results may be limited to expository passages that describe how concrete physical, biological, or social systems work rather than descriptive or narrative passages” [48].

Thus, CMI is more aligned with Mayer and Anderson’s [48] notion of arbitrary facts that cannot be tested for understanding and are more descriptive than expository in nature. Thus, it is not unreasonable to assume that no differences were observed in memory because CMI is poorly suited for gains associated with multimedia instruction, but this does not necessarily apply to other types of consumer health information.

#### Multimedia Benefits Some More Than Others

No gains in memory in this study may be attributable to participants being younger and/or having adequate health literacy. The participants in this study were younger, well-educated, and had adequate health literacy and eHealth literacy. One or all of these sample characteristics may have limited the potential benefits of multimedia presentation of health information or specifically CMI in this study.

Multimedia may be more beneficial for older people than for younger people. Many older people are affected by a decline in one or more cognitive capabilities, which can create negative implications for learning [53,54]. Age-related cognitive decline includes reductions in processing capacity, cognitive speed, inhibition, coordination, and integration [54]. However, the cognitive aging principle [53] asserts that the application of multimedia strategies can help older learners overcome obstacles due to age-related limitations in cognitive capabilities. Some studies have found more pronounced benefits (ie, interactions) of multimedia instruction for older people than for younger people [53,55]. Thus, the benefits of multimedia instruction for CMI may only apply to older adults. However, the evidence is mixed, as other studies have found that both younger and older people benefit equally from multimedia instruction [54,56], suggesting that despite the younger sample in this study, benefits due to multimedia instruction should still have been observed.

Benefits due to multimedia instruction may be more pronounced for people with limited literacy than for those who have adequate literacy. In a review of 55 studies comparing text alone with illustrated text, Levie and Lentz [57] found that there was some evidence to support the argument that illustrations are more helpful for poor readers than for adequate readers. Further, in their review, Houts and colleagues [21] reported that people with low literacy levels were more likely to benefit from multimedia instruction in consumer health information. Although literacy itself was not measured in this study, the high levels of health literacy and education in this sample likely precludes these participants from having literacy issues. Thus, the current sample may not have benefitted from multimedia instruction because of their adequate levels of literacy.

#### Multimedia Improves Perceptions and People Prefer it

Interestingly, although objectively all 3 formats were nearly equivalent in terms of memory, participants did perceive the formats differently and preferred one multimedia format overall. Specifically, participants perceived the text + images format to be more comprehensible and to have higher design quality than the other 2 formats. Additionally, there was some evidence that participants perceived the text + images format to have more utility, but this finding was not robust enough to be significant after correcting for multiple comparisons. It would have been most surprising if the utility of any of the formats was perceived differently because the content was held constant between the 3 formats. Consistent with the participants’ perceptions, most participants chose the text + images format as their most favorite overall.

In contrast to the findings from this study, a previous study found that multimedia medication information impacted only the likelihood that people would refer to the handout in the future but not its ratings of user-friendliness, long-term comprehension, or effectiveness [58]. No demographic information (eg, socioeconomic status, ethnicity, age) was collected from their sample. However, based on population statistics, Advani and colleagues [58] posited that their inconclusive results on multimedia medication information preferences might be due to a sample of participants with potentially high levels of health literacy who appeared to appreciate text-only materials. However, our sample had adequate to high levels of health and eHealth literacy, which would suggest that other factors (eg, age, technology use) might be more predictive of whether or not people perceive multimedia medication information more favourably than strictly text-based materials. However, we cannot determine with any certainty what motivated these differences without additional research.

### Limitations

There were several limitations that may affect the transferability and generalizability of the results of this study. Opinions and performance of young, educated, generally healthy adults, such as those in this sample of participants, may not be representative of other groups of consumers, or consumers as a whole. This study used a convenience sample, which resulted in a predominantly female sample who had higher than expected rates of prescription medication use compared with national averages [3]. Additionally, due to the stimuli exclusion process to enhance equivalency, all pills were excluded. However, pills are likely the most frequently prescribed, dispensed, and used medications. The NVS [39] has only been validated using paper administration, not online administration as in this study. Further, subscales from the adapted CIRF [42] inspired the single-item perception measures of comprehensibility, utility, and design quality. However, collapsing multiple ratings into single-item measures resulted in them being inherently less detailed and made it difficult to determine with any certainty to what extent individual factors influenced these perceptions. Finally, given the time limitations, participants were only exposed to the information in the narration + images condition once verbally, whereas—depending on their reading rates—they may have been able to revisit information in the other 2 conditions (ie, text, text + images) more than once.

### Conclusions and Future Directions

There are several valuable conclusions to be drawn from this study. Like other consumer health information, effort has been exerted to develop CMI and human resources are continuously invested into dispensing them to consumers in hope that they will help educate people on the benefits and minimize the potential consequences of risks associated with medications. However, merely providing materials to consumers does not ensure that they will use them and indeed usage rates of medication information tend to be low. For example, medication information reading rates in a similar sample of university students (N=306; mean age 23.6 years) found that 37% of participants reported reading CMI always or often, and an alarming 32% participants reported reading it rarely or never [28]. Thus, if making these materials more appealing to users increases the likelihood of them being used, that would be a worthwhile investment. Therefore, it would be worthwhile to investigate whether incorporating multimedia into CMI has a positive impact on reading rates of these materials.

CMI also creates challenges around disentangling comprehension from memory. Future work would benefit from determining methods to examine comprehension independently from memory and information localization. Arguably, memory is important in circumstances when CMI is unavailable, whereas information localization and comprehension take precedence when CMI is available. Unfortunately, the current practice of distributing CMI as a hard copy often renders them unavailable. However, this situation will likely be remedied when digital methods of CMI distribution are adopted.

This study focused on only a narrow aspect of eHealth literacy competencies, but other facets of eHealth literacy could be explored using CMI. Specifically, we developed this experiment on the premise that consumers received medication information, as is currently the typical practice in North America. Therefore, the focus on this study was whether participants would remember different aspects of the information to simulate addressing or solving a health problem (eg, experiencing a side effect, missing a dose) rather than the acts of seeking, finding, and appraising health information from electronic sources. Thus, there are many other aspects of citizens’ actual medication information use that warrant exploration. For example, do people use paper copies of CMI or online resources instead? What online resources do citizens prefer? If CMI was digitized, how would citizens like to receive it (eg, in a mobile app, on a pharmacy website, by email)? Moreover, at what point in the prescription process would citizens want digital CMI? What factors would impact the usage rates and efficacy of digitized CMI?

Although multimedia is a potentially valuable tool for consumer health information, the conditions in which benefits are observed may be limited to specific people, specific stimuli, or other specific contexts. For example, in this study with a sample of younger, adequately health literate people, no improvements in memory for a specific type of health information (ie, CMI) were observed. That does not preclude benefits of multimedia for other types of multimedia consumer health information for older people and/or people who have limited health or eHealth literacy, who may arguably be helped more by multimedia materials. Moreover, despite the lack of objective improvements as a result of multimedia, subjective improvements (ie, peoples’ perceptions and preferences) for multimedia CMI were significantly enhanced. Although ideally we would have observed improvements in both subjective and objective measures, we cannot discount the importance of peoples’ opinions of consumer health information. Multimedia consumer health information warrants more investigation with respect to what impacts it has on which specific subjective and objective measures and under what conditions (eg, stimuli topics, characteristics of the sample). If evidence suggests that performance and perceptions of certain groups of people are affected variably by multimedia information, it may further motivate argument for tailored health information that aligns with individuals’ information needs.

## Appendix

Multimedia Appendix 1 Sample of the text format.

Multimedia Appendix 2 Sample of the text + images format.

Multimedia Appendix 3 Sample of the narration + images format.

Multimedia Appendix 4 Experimental tasks.

## Abbreviations

ANOVA: analysis of variance
CIRF: Consumer Information Rating Form
CMI: consumer medication information
CTML: cognitive theory of multimedia learning
DIN: drug identification number
eHEALS: eHealth Literacy Scale
NVS: Newest Vital Sign
