# Supplementary material for: Differences in Memory, Perceptions, and Preferences of Multimedia Consumer Medication Information: Experimental Performance and Self-Report Study
Source: JMIR Hum Factors. 2020 Dec 1;7(4):e15913. doi: 10.2196/15913 (PMC7738255; doi:10.2196/15913)
Supplement: Multimedia Appendix 1 [file humanfactors_v7i4e15913_app1.docx]

Content developed by a leading Canadian pharmacy.

**Brand Name:** CROMOLYN OP DRP 2%

**Chemical Name:** SODIUM CROMOGLYCATE 2%

**Common uses**

This medication is typically used for allergic conjunctivitis (inflammation of the clear covering of the front of the eye, due to allergies). It requires several days to take effect.

**How to use this medication**

This medication is for the eye. To use:

- wash your hands as well as the skin surrounding the affected eye;
- shake the container;
- gently pull down the lower lid of the affected eye;
- apply 1 drop of the medication;
- close the eye for 2 to 3 minutes, while gently pressing on the inner corner of the eye.

To avoid contaminating the medication, do not let the tip of the applicator touch your fingers or any part of your eye. Close the container tightly after each use.

This medication is typically used 4 times a day. However, your doctor or pharmacist may have suggested a different schedule that is more appropriate for you. Use it regularly and continuously to maintain its beneficial effects.

If you forget a dose, apply it as soon as you remember – unless it is almost time for your next dose. In that case, skip the missed one. Do not apply more to try to catch up.

**Possible side effects**

In addition to its desired action, this medication may cause some side effects, notably:

- when it is used, it may cause local irritation
- when it is used, it may cause a temporary burning sensation
- it may cause a stinging sensation.
