# Supplementary material for: Differences in Memory, Perceptions, and Preferences of Multimedia Consumer Medication Information: Experimental Performance and Self-Report Study
Source: JMIR Hum Factors. 2020 Dec 1;7(4):e15913. doi: 10.2196/15913 (PMC7738255; doi:10.2196/15913)
Supplement: Multimedia Appendix 2 [file humanfactors_v7i4e15913_app2.docx]

Written content developed by a leading Canadian pharmacy.


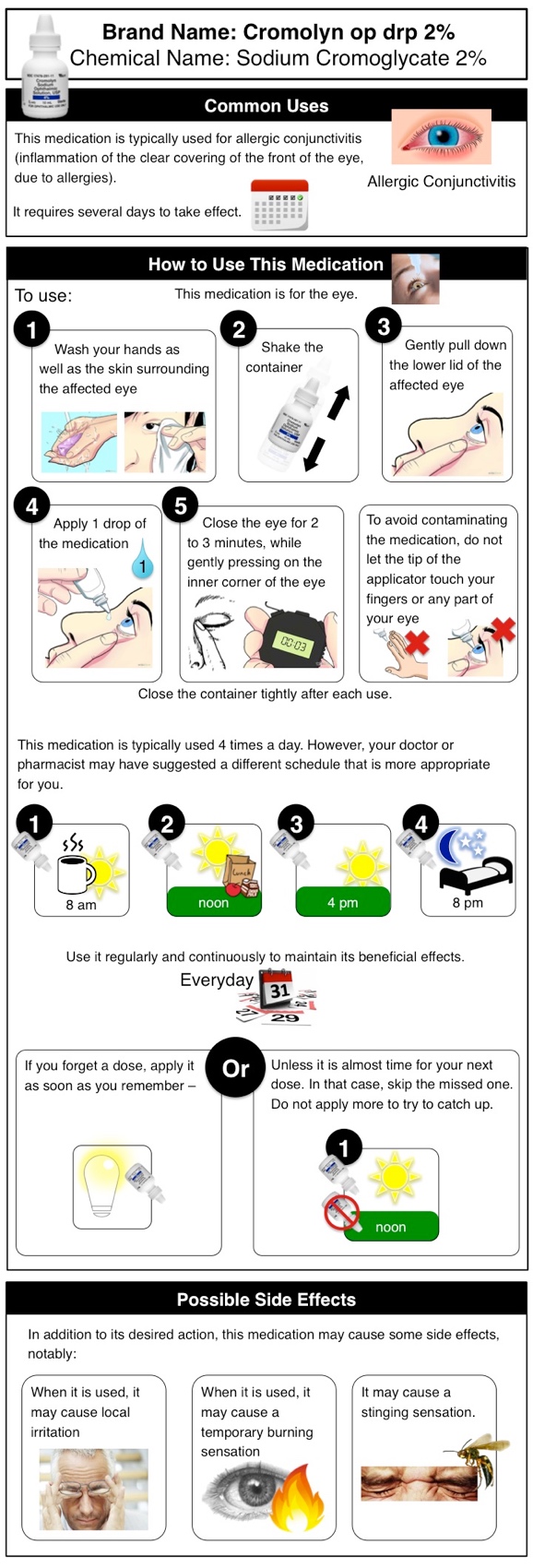


### Image Credits for Cromolyn

The following images were used to create the Text + Images & Narration + Images conditions, from left to right, top to bottom:

Akorn. Cromolyn Sodium Opthalmic Solution, USP. “Cromolyn bottle”. Retrieved from <http://www.akorn.com/prod_detail.php?ndc=17478-291-11>

Dawson Health Office News. *Pink eye/conjunctivitis.* “Allergic conjunctivitis”. Retrieved from

<https://sites.google.com/a/wrsd.net/dawson-health-office-news/health-conditions/pink-eye-conjunctivitis>

Kennebec Valley Community College. *Kennebec Valley Community College Campus events.* “Calendar”. Retrieved from <http://www.kvcc.me.edu/pages/student-life-development/campus-events>

Search Home Remedy. *Rose water eye drops.* Retrieved from <http://www.searchhomeremedy.com/rose-water-eye-drops/>

“Handwashing” by <https://www.wikihow.com/> is licensed under Creative Commons [CC BY-NC-SA 3.0 License](http://creativecommons.org/licenses/by-nc-sa/3.0/).

“Eye washing” by <https://www.wikihow.com/> is licensed under Creative Commons [CC BY-NC-SA 3.0 License](http://creativecommons.org/licenses/by-nc-sa/3.0/).

Akorn. Cromolyn Sodium Opthalmic Solution, USP. “Cromolyn bottle”. Retrieved from <http://www.akorn.com/prod_detail.php?ndc=17478-291-11>

“Pull down lower eyelid” by <https://www.wikihow.com/> is licensed under Creative Commons [CC BY-NC-SA 3.0 License](http://creativecommons.org/licenses/by-nc-sa/3.0/).

“Pull down lower eyelid” by <https://www.wikihow.com/> is licensed under Creative Commons [CC BY-NC-SA 3.0 License](http://creativecommons.org/licenses/by-nc-sa/3.0/).

“Insert eye drop” by <https://www.wikihow.com/> is licensed under Creative Commons [CC BY-NC-SA 3.0 License](http://creativecommons.org/licenses/by-nc-sa/3.0/).

Health Park Pharmacy. Eye health: Dry eye & eye drops. Retrieved from <http://healthparkpharmacy.com/eye-health-2/>

Clipart Panda. *Kids lunch time clipart: Viewing Gallery For - Clip Art.* Retrieved from <http://www.clipartpanda.com/clipart_images/viewing-gallery-for-clip-art-38820357>

“Man with glasses rubbing eyes”. Unavailable.

DRAWING THE HUMAN EYE ONLYPENCIL DRAWING TUTORIALS. Retrieved from <https://drawingpenciled.com/item/136037/drawing-the-human-eye-onlypencil-drawing-tutorials-pencil-eye-drawing-tutorial.html>

Llama Cartoon Gratis PNG y Clipart. Retrieved from <https://es.pngtree.com/freepng/flame-cartoon_3431574.html>

THE VALUE OF PAIN: LEPROSY. Retrieved from <http://motionstability.com/the-value-of-pain-leprosy/>

Purchased from iStock by Getty Images. Monster Wasp – Stock Image. Retrieved from <https://www.istockphoto.com/gb/photo/monster-wasp-gm139262382-322068>
