# Supplementary material for: Differences in Memory, Perceptions, and Preferences of Multimedia Consumer Medication Information: Experimental Performance and Self-Report Study
Source: JMIR Hum Factors. 2020 Dec 1;7(4):e15913. doi: 10.2196/15913 (PMC7738255; doi:10.2196/15913)
Supplement: Multimedia Appendix 4 [file humanfactors_v7i4e15913_app4.docx]

**Multimedia Appendix 4 - Experimental Tasks**

**Memory Task**

The lead investigator introduced the task by saying “I will now ask you questions about the medication you just learned about. Please do your best to remember as much as you can. Some of the questions are not applicable to all of the medications. If you think the question is not applicable, please tell me and we will skip it.”

Then she posed the following questions to participants:

1. What were the two names of the medication?
2. Why would you be taking this medication? That is, what condition or conditions is the medication usually used to treat?
3. How long does it typically take for the medication to start working?
4. Where do you apply or administer this medication?
5. How would you take this medication? That is, what are the instructions and steps?
6. How many times a day is this medication usually taken?
7. What should you do if you missed a dose of this medication?
8. What are the potential side effects of this medication?
9. Is there any other important information to remember about this medication?
10. Is there anything else you remember about this medication?
11. *(Additional explanatory question) If someone didn’t know anything about this taking this medication, what would you tell them?

**Perceptions Ratings**

Participants rated each of the three formats (i.e., Text, Text + Images, Narration + Images) on a on a 5-point scale from very poor to very good for each of the following questions (i.e., nine ratings in total).

1. Overall, how would you rate the **comprehensibility** of each format?

Comprehensibility: Easy to read/hear, understand, remember, locate important information, keep for future reference

1. Overall, how would you rate the **utility** of each format?

Utility: how appropriate (not too much or too little) was the amount of information and how useful was the information about the medication benefits, precautions, instructions, warnings, side effects, etc.

1. Overall, how would you rate the **design quality** of each format?

Design Quality: organization attractiveness, print size, tone, helpfulness, bias, line spacing

**Preference Ranking**

Following rating each format on each perception dimension, participants ranked the three formats from favourite, middle, to least favourite format, ties were impossible.
